# Preparation of a Selective L-Phenylalanine Imprinted Polymer Implicated in Patients with Phenylketonuria

**Published:** 2014-11

**Authors:** Parvaneh Najafizadeh, Soltan Ahmad Ebrahimi, Mohammad Reza Panjehshahin, Seyed Mahdi Rezayat Sorkhabadi

**Affiliations:** 1 Department of Pharmacology, Pharmaceutical Sciences Branch, Islamic Azad University, Tehran, Iran;; 2 Department of Pharmacology, Faculty of Medicine, Iran University of Medical Sciences, Tehran, Iran;; 3 Department of Pharmacology, School of Medicine, Shiraz University of Medical Sciences, Shiraz, Iran;; 4 Department of Pharmacology, Faculty of Medicine, Tehran University of Medical Sciences, Tehran, Iran

**Keywords:** Phenylalanine, Phenylketonurias, Treatment

## Abstract

**Background: **Molecular imprinting is a method for synthesizing polymers with structure-selective adsorption properties with applications such as, selectivity binding, drug delivery systems and anti-bodies. The present study aims at optimizing the preparation of molecularly imprinted polymer (MIP) against l-phenylalanine, in order to increase phenylalanine-binding in Enzymatic Intestinal Simulated Fluid (ESIF).

**Methods:** The MIP for l-phenylalanine, as a water-soluble template, was successfully synthesized without derivatization. Synthesization was done by a UV polymerization method in which methacrylic acid (MAA), as a functional monomer, and ethylene glycol dimethacrylate (EGDMA), as a cross-linker, were used in the presence of five different porogenic solvents including; acetonitrile, tetrahydrofuran (THF), chloroform, toluene and dimethyl sulfoxide (DMSO). The selectivity of the MIP was examined using 19 different amino acids in human serum and was evaluated by HPLC. In addition, morphological studies were conducted using SEM.

**Results:** The results showed that the obtained MIP with acetonitrile had the highest capacity and selectivity compared with other solvents. The data indicated that Phe-binding to MIP was significantly more than the former binding to NIP in EISF (P≤0.05). Moreover, in comparison with NIP and control group, MIP showed a better selectivity and binding for Phe. This could be used for the reduction of Phe in human serum samples of Phenylketonuria.

**Conclusion: **Our findings suggest that the MIP against Phe prepared with acetonitrile, showed a good selectivity and binding, which caused a reduction of blood Phe concentration in enzymatic simulated intestinal fluid and human serum sample of Phenylketonuria.

## Introduction

Molecular imprinting is a new and practical technique, which leads to the preparation of selective recognition sites in a polymer matrix. Molecularly imprinted polymers are prepared by co-polymerization of a cross linking agent with the formed complex from a template and a functional monomer.^1^^,^^2^ The removal of the template molecule by extraction leads to the obtained polymer matrix which produces vacant recognition sites that exhibit recognition ability and have a pre-designed selectivity to the template molecule and structurally related compounds.

The MIPs might then be used as an artificial receptor to selectively rebind the template from a mixture of chemical species.^3^ This technique has a variety of applications including, chiral stationary phases for chromatography, biomimetic sensors, separating material and drug delivery systems.^4^^,^^5^

Phenylalanine (Phe) is an essential amino acid, which is necessary for infants’ growth and nitrogen equilibrium in adults. However, phenylalanine’s accumulation could cause neurological damages which are the results of deficiency in phenylalanine hydroxylase enzyme, considered as the major enzyme for its metabolism.^6^^,^^7^ This enzyme typically begins the procedure of parsing of phenylalanine amino acid molecules which is essential for protein synthesis in the body. Mutations in the phenylalanine hydroxylase gene will cause a disease called phenylketonuria (PKU).^7^ PKU is an autosomal and recessive disease with symptoms such as behavioral abnormalities, motor dysfunctions and mental retardation which is associated with impaired brain development. CNS symptoms of this disorder include microcephaly, epilepsy, and severe intellectual disability. Although there is no cure, PKU can be treated with a carefully regulated diet, as well as with close monitoring of Phe blood levels.^8^ With such treatment, it is not uncommon for individuals afflicted with PKU to avoid negative accumulative Phe side effects and live a normal life. Additionally, dietary restriction of phenylalanine remains the mainstay of the treatment for PKU, which is still a potential area of research and development for new treatments.^9^^-^^11^

Regarding MIP’s applications and characteristics, especially high selectivity for specific molecules, and since PKU treatments are aimed at decreasing Phe concentration in the blood, the purpose of this study is to synthesize and optimize the phenylalanine imprinted polymer. This is done with different solvents in order to decrease its concentration in an enzymatic simulated intestinal fluid. Furthermore, it is intended to investigate the selectivity of L-Phenylalanine imprinted polymer for the reduction of phenylalanine concentration in human PKU plasma. 

## Materials and Methods

*Chemicals and Materials*

L-Phenylalanine, methacrylic acid (MAA), ethylene glycol dimethacrylate (EGDMA), trichloroacetic acid (TCA), trifluoroacetic acid (TFA), acetonitrile, toluene, tetrahydrofuran (THF), dimethyl sulfoxide (DMSO), chloroform, ortho phenyl aldehyde (OPA), N-methyl-L-phenylalanine, potassium phosphate monobasic (KH2PO4), pancreatin and methanol were purchased from Sigma-Aldrich (Steinheim, Germany). 2-2′-azobisisobutyronitrile (AIBN) was purchased from Nippon Shiyaku, Inc. (Tokyo, Japan). All other chemicals were of ANALAR grade and all solvents were of HPLC grade. Serum samples for the experiments were received from Dr. Ebrahimi, Masoud Laboratory (Tehran, Iran).

*Preparation of Molecularly Imprinted Polymers for L-phenylalanine with Different Porogenic Solvents*

Molecularly Imprinted Polymer for L-phenylalanine was prepared by a UV polymerization method as follows:

Two solutions were prepared; solution (1) was prepared by mixing L-phenylalanine (0.16 gr), MAA (0.34 ml), 3 ml porogenic solvent (e.g. toluene: P1), acetic acid (0.6 ml) and TCA (0.19 gr). The mixture was sonicated for 10 minutes. Solution (2) was prepared by adding AIBN (0.19 gr) and EGDMA (3.96 ml) which were dissolved in 2 ml porogenic solvent (toluene: P1). Then, the solutions were mixed in a glass tube and stirred for 5 minutes. The resulting solution was sparged with argon for 5 minutes and the sealed tube was put under UV light for 24 hours. This process was repeated with different porogenic solvents of acetonitrile, THF, chloroform and DMSO labeled with sample numbers of P2, P3, P4, and P5.

The ground polymer was washed with 300 ml methanol (4 times) and with 100 ml TCA 10% (once). The polymer was then re-suspended in 500 ml 5% v/v acetic acid and sonicated for 10 minutes. The polymer was repeatedly washed with distilled water to remove the template completely until pH of polymer suspension and distilled water became the same. Furthermore, the binding percentage and selective performance of the MIPs were assessed through HPLC analysis. Among the polymers used, the best with the most binding percentage was selected for the next phase of the study.^12^

*Batch Adsorption Procedure in Enzymatic Simulated Intestinal Fluid*

50 mg of MIP or NIP was incubated with 50 µmol of phenylalanine in 1.5 ml of enzymatic Simulated Intestinal Fluid (USP 31, 2008), at 37°C. At timed intervals, 75 µL of the reaction mixture was taken and centrifuged (at 14000 rpm; 5 min). Then, 50 µL of supernatant was injected to HPLC until the equilibration time was reached.

Having the initial concentration of phenylalanine and knowing the number of moles in the solution phase from the beginning of the reaction, the number of remaining moles after equilibration was calculated and subtracted from the initial amount to gain the number of bounded moles to the polymer.

*HPLC Condition for Measurement of Phe in Enzymatic Intestinal Simulated Fluid*

Chromatographic determination of components was carried out on the HPLC system and consisted of a 9000 pump (Younglin, Korea), a UV-Vis detector (Younglin, Korea), a manual injector (Rheodyne, USA) and system control software (Autochro-2000, Younglin, Korea). The analytical column (250X 4.2 mm, Tracer Excel C18) was eluted with methanol:water:TFA (30:69.9:0.1) at a flow rate of 1 ml per min. The wavelength used for detection was 210 nm. 

*Adsorption Experiments for the Selectivity of the Phenylalanine Imprinted Polymer in Different Serums.*

Initially, two plasma samples (1 ml) were taken from two different cases with PKU as well as a healthy person. Then, 300 µl of each of these samples was incubated, every time, with 10 mg dry NIP, MIP and with a control group (a group without MIP or NIP) at 37°C for 180 minutes. For quantification of the total and individual amino acids (Histidine, Alanine, Tyrosine, Valine, Methionine, Phenylalanine, Leucin, Isoleucin, Serine, Aspartic acid, Glutamine, Lysine, Arginine, Glycine, Threonine, Aspargine, Ornithine, Citruline, Glutamic acid) estimated in each serum, the derivatization method was deployed as briefly described hereafter. After denaturing the plasma’s protein by methanol extract (800 μl), 100 µl supernatant of borate buffer was added to each of the tubes, followed by 50 µl OPA reagent (2 min) and 25 µl of 0.25 MHCl which were added to each of the tubes and then mixed. Finally, 250 µl of the mobile phase was added and 20 µl of the resulted solution was injected to HPLC.^13^^,^^14^

*HPLC Condition for Measurement of Amino Acids in Human Serum*

Chromatographic determination of components was carried out on a HPLC system consisting of a 9000 pump (Younglin, Korea), a fluorescent detector (Waters, USA), a manual injector (Rheodyne, USA), system control software (Autochro-2000, Yong Lin, Korea) and analytical column (250X 4.2 mm, Tracer Excel, C-18). The elution of derivatized amino acids was effected by a gradient of two mobile phases: (A) sodium acetate (50 mM, pH 7):methanol:THF (59:32:9) and (B) sodium acetate (300 mM, pH 5):methanol:THF (22:77:1). The mobile phase flow rate was set at 1.3 ml per minute. Initial mobile phase composition of 100% (A) was changed linearly to 100% (B) after 35 min. The excitation was set to 330 nm and emission at 450 nm was recorded. 

*Morphological Study of MIP or NIP Using Scanning Electron Microscope*

Scanning electron microscope (SEM) model VEGA-TESCAN-LMU was used to visualize the surface features of the polymer. SEM specimens were prepared by redispersing the microspheres in ethanol and placing a drop on a piece of cover glass, which was mounted on an aluminum stud. After solvent evaporation, the particles were sputter-coated with a thin layer of gold.

*Statistical Analysis*

The data presented as mean±SEM were analyzed using student’s t test and one-way Analysis of Variance (ANOVA). Where a significant difference was obtained with one-way ANOVA, the source of the difference was located using Tukey test. The data were analyzed using SPSS. P≤0.05 was statistically significant. 

## Results

*Effect of Porogenic Solvent on Selective Efficiency of Molecularly Imprinted Polymer for L-Phenylalanine*

**Figure 1:**
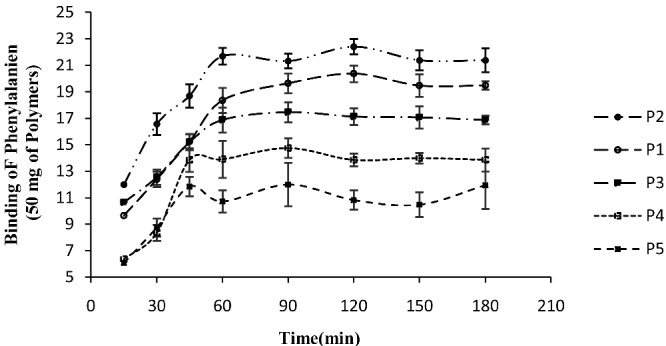
Binding of Phe to 50 mg of the MIPs with different solvent at different times (n=3). Each point represents mean±SEM; Phe concentration in EISF was 50 µg/ml.

Figure 1 shows the batch adsorption of MIPs with different porogenic solvents for Phe at different time intervals. The order of protogenic solvents on adsorption efficiency was acetonitrile>toluene>THF>chloroform>DMSO.

The adsorptions were initially fast during the first 45 minutes and then slowed down. After 90 minutes, assumed adsorption equilibrium was achieved.

*Effect of Incubation Time on Phe Binding to MIP and NIP in Enzymatic Intestinal Simulated Fluid*

**Figure 2:**
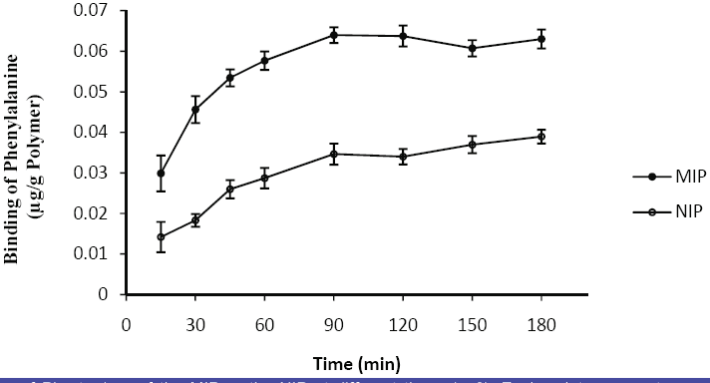
Binding of Phe to 1 gr of the MIP or the NIP at different times (n=3). Each point represents mean±SEM; Phe concentration in EISF was 50 µg/ml.

Figure 2 shows the changes in Phe binding to either MIP or NIP with time. It is shown that about 80% binding of Phe to MIP acquired within 90 minutes during which a maximum binding was reached. The Phe binding curve of NIP presented a similar profile. Based on the observations, 180 minutes was the best time for the attainment of equilibrium for Phe-binding to MIP and NIP. In subsequent experiments, 180 minutes also permitted equilibration between polymer and Phe. Phe-binding to MIP was significantly more than NIP (P=0.04).

*Effect of Adsorption of MIP and NIP on Different Human Serums*

**Figure 3:**
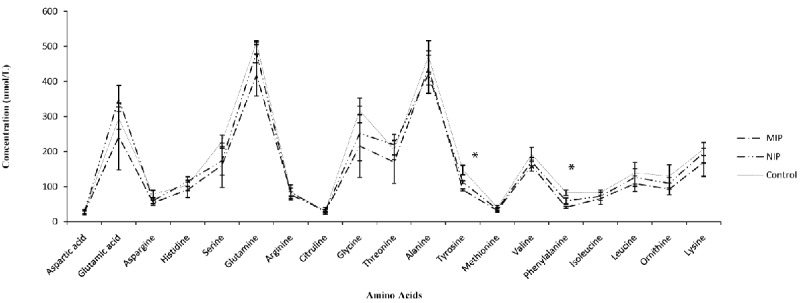
Binding of different amino acids to MIP or NIP in normal serum.  Each point represents mean±SEM. (n=3).

**Figure 4:**
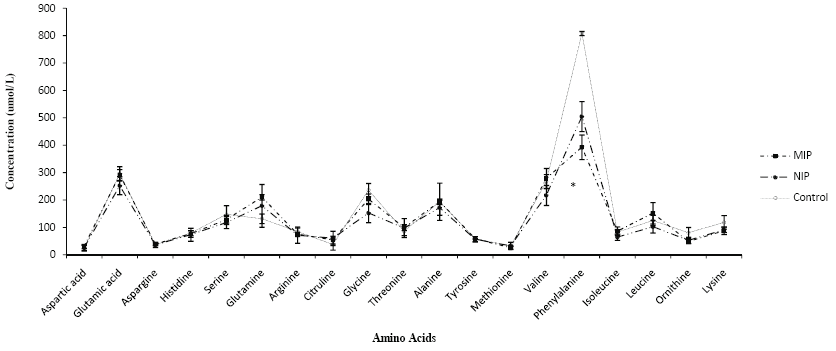
Binding of different amino acids to MIP or NIP in PKU serum.  Each point represents mean±SEM. (n=3).

Figure 3 and 4 show the effects of treatment of healthy and PKU serums with either MIP or NIP. Figure 3 indicates that both MIP and NIP have significantly decreased the amount of Phe (P=0.03) and tyrosine (P=0.04) in healthy serum. In figure 4, MIP has significantly reduced Phe concentration compared with NIP and control group with PKU serum. (P=0.02)

*SEM Analysis*

**Figure 5:**
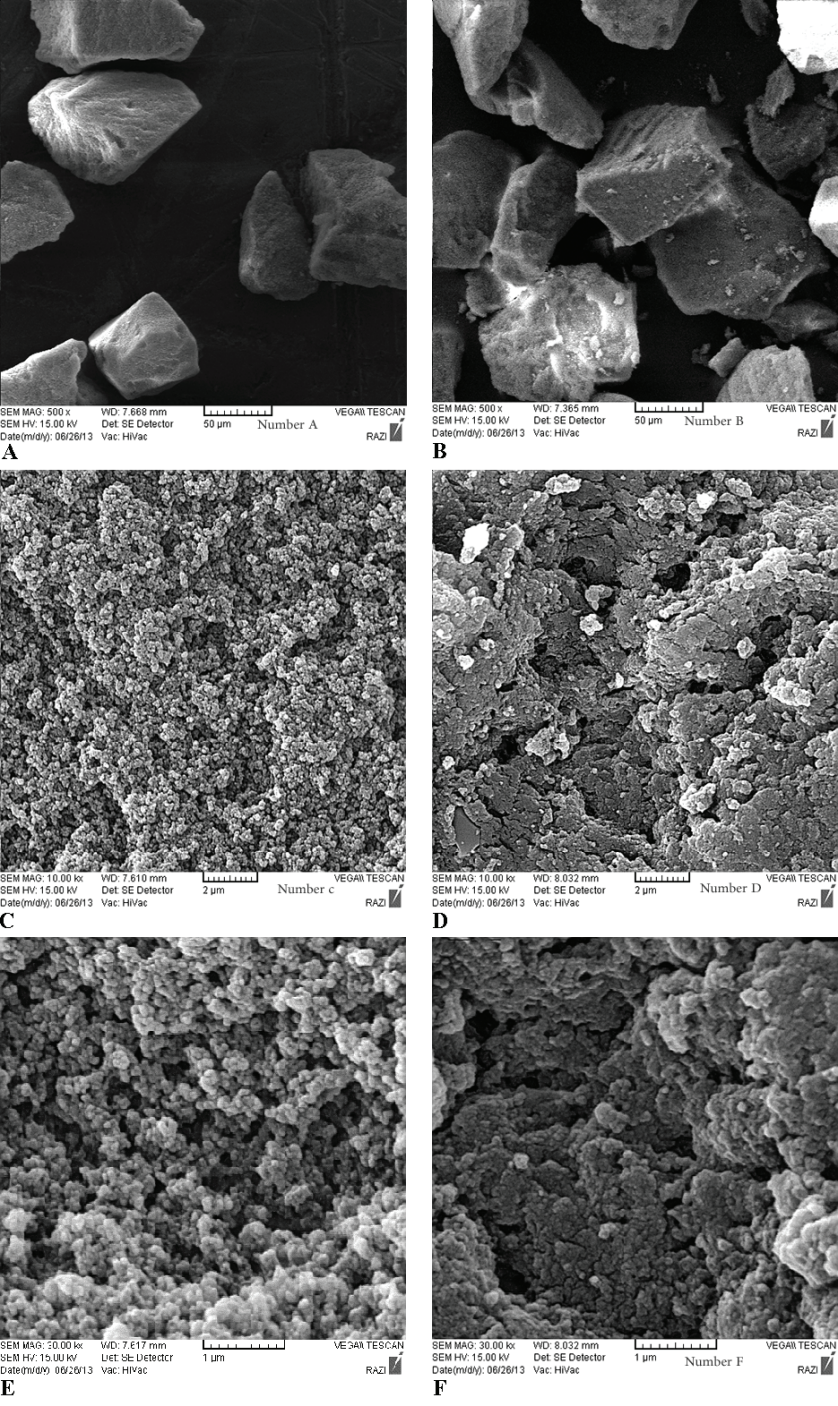
SEM micrographs of polymer (B, D, F) non-imprinted and (A, C, E) imprinted with Phe as the molecule template

Figure 5 is a scanning electron micrograph showing the morphology or structure of the Phe-imprinted polymer of MIP and NIP.

The images show appreciable differences in the morphology of the polymer. The NIP has more uniform and smooth shape than the MIP, which has an irregular and rough morphology (rather like micro-particles with small cavities). There are many microspores inlaid in the network skeleton of Phe-MIP, which indicates the creation of a space for binding of Phe. 

## Discussion

The main objective of the present study was to examine the synthesis of an imprinted phenylalanine polymer with different porogenic solvent useful in lowering Phe-concentration, the high amount of which serves as the main cause of PKU. The findings showed that the imprinted polymer of Phe with acetonitrile could decrease Phe-concentration in enzymatic Simulated Intestinal Fluid. 

In the first study of an experimental preparation of Phe imprinted polymer, MAA was used as a functional monomer. In one hand, due to the presence of one amine group, Phe can be easily bound to this acidic functional monomer. On the other hand, the presence of carboxyl group in MAA can form hydrogen bond, which produces recognition sites. Similarly, MAA has been used in other studies,^15^^,^^16^ but in a study by Qiu et al., the mixture of Acrylamide and MAA was used for MIP preparation.^17^

Phe is a non-polar amino acid with a hydrophobic nature of the benzyl side chain which is only soluble in water and slightly in Ethanol.^18^ Due to the number of problems posed by the fundamental differences between two types of organic (Van der Waals interactions) and aqueous media systems (hydrophobic interactions), water was not used as a solvent in this study. Instead, in the present study, acetonitrile and toluene, THF, DMSO, chloroform as porogen solvents were used. Different porogen solvents play an essential role in the strength of interaction between MAA (functional monomer) and EGDMA (cross linker). In addition, porogen solvent generates pores that enhance the recognition sites and influences the morphology of MIP.^19^ Song et al. examined the effect of porogenic solvents on querecetin and showed that THF had the best effect, even though the study conditions, template and functional monomer type were different.^20^ Shah et al. and also Lee et al. synthesized an MIP for Phe with toluene as a porogenic solvent.^15^^,^^16^ There has been no study, until now, which has investigated the effects of different porogenic solvents in Phe-MIP.

Studies about the treatment effect of MIP and NIP on human serum show that MIP decreases Phe concentration. However, it is possible that they have the same effect on the other amino acids, which could be for their non-selective binding to MIP or NIP. In these studies, the rate of binding capacity to MIP for Phe was more than NIP and the rate of different amino acids binding to NIP indicates the non-selective binding to NIP. 

SEM studies indicate that the good morphologic properties for the MIP were more likely originated from the polymerization process. There are at least three factors that should be taken into account, the polymerization temperature, the composition of pre-polymerized solution (e.g. the type and amount of crosslinker) and the porogenic solvent, which are related to the adsorption process. The regular structure of NIP was resulted, as there were no selective binding sites created for the analysis. The cavities in the MIP were probably caused by the target molecule structure (phe).^21^

## Conclusion

MIP against Phe was prepared by UV polymerization method using Phe as template molecule, MAA as a functional monomer and EGDMA as a cross-linker in the presence of five different porogenic solvents. The results indicate that the type of porogenic solvents had a major impact on the formation of MIP and its Phe adsorption. The best solvent was acetonitrile and the worst was DMSO. The findings suggest that the MIP preparation for Phe can decrease Phe-concentration in EISF. However, further studies are needed to investigate the increase of binding capacity in MIP that might be used in future treatments of PKU. 
